# Ultrasensitive analysis of genetic instability related to chemical exposure

**DOI:** 10.1007/s13353-021-00677-6

**Published:** 2021-12-30

**Authors:** Tomasz Domoradzki, Piotr Grochowski, Anna Jaśkiewicz, Beata Pająk

**Affiliations:** 1 Kaczkowski Military Institute of Hygiene and Epidemiology, Kozielska 4, 01-089 Warsaw, Poland; 2 Kawaska Sp. z o. o., Zaczarowanej Róży 1, 05-540 Zalesie Górne, Poland

**Keywords:** Ammunition, Toxic chemical compounds, Long-term exposure, Genetic analysis, SNPs, Lung carcinogenesis

## Abstract

Our concerns have been raised about whether prolonged exposure to ammunition-related chemicals could correlate with genomic instability predisposing to lung carcinogenesis. The group of professional soldiers engaged in routine ammunition analysis and its explosive properties testing. To assess the presence of an innate genetic profile, DNA isolated from swabs was analyzed with LungCarta and HS Lung Panels and MassARRAY Analyzer 4 mass spectrometry. The presence of new somatic single nucleotide polymorphisms (SNPs) was detected with liquid biopsy technique and circulating cell-free DNA (ccfDNA) isolation, which was further analyzed with UltraSeek Lung Panel. Both genetic profiles obtained for each individual were compared. Genetic analysis revealed that in professional soldiers with long-term exposure to ammunition-related toxic chemicals, new SNPs in genes related to lung carcinogenesis are present. The preliminary data indicate that using modern molecular techniques could be a valuable tool for monitoring the genome instability in context of occupational risk of harmful volatile toxic compounds and improving personnel safety. The analyzed group will be further enlarged, and follow-up studies will be continued.

**Supplementary Information:**

The online version contains supplementary material available at 10.1007/s13353-021-00677-6.

## Introduction

One of the critical research tasks to assess the adverse health effects of exposure of professional soldiers to chemical, physical, and biological agents in the place of service is implementing modern molecular diagnostic methods and identifying threats that could predispose to their occurrence. Molecular diagnostics of DNA integrity and stability, enriched with the assessment of proteome homeostasis and inflammatory markers, allows for early detection of adverse changes at the cell level. It could allow preventive actions, such as reducing exposure, changing work position, or preventive treatment (Jackson and Bartek 2009). It is well known that decreased genome integrity, accumulation of point mutations, chromosomes translocations, aneuploidy and polyploidy, telomere shortening, abnormal protein maturation processes, and finally, high levels of oxidative stress are the cause of neoplastic, neurodegenerative, and metabolic diseases (Feltes et al. 2016). The introduction of tools for assessing and monitoring molecular health markers could significantly improve the identification of highly harmful exposure factors and identify soldiers who should be subject to detailed medical supervision.

### Justification for the selection of the exposure factor and the study group

The specificity of the work of soldiers and the impact of a wide range of factors: ionizing and electromagnetic radiation, toxic chemical agents, and various biological factors, may generate more rapid changes in the organization of genetic information than among people of similar age, not exposed to harmful environmental conditions. It is well known that the negative impact of environmental conditions determines the appearance of point mutations and the accumulation of which leads, among others, to the development of neoplastic diseases of various organs (Ackerman and Horton 2018). In the presented research, using the conducted at the Kaczkowski Military Institute of Hygiene and Epidemiology catalog of harmful and carcinogenic substances present in the workplaces of soldiers, engaged in the experimental characterization of explosives (under National Health Program 2016–2020), we identified and assessed the effect of selected chemical factors on genomic stability, examined by the presence of point mutations in a selected set of genes. Characterization of explosives includes synthesis process, chemical and physical properties assessment, stability and sensitivity analysis, formulation, detonation parameters, and explosive effects. Chemical factors that have received our particular attention are the products of ammunition combustion, recognized previously as harmful for human health by inhalation (e.g., diphenylamine, carbon monoxide, HC hydrocarbons and their derivatives defined as volatile organic compounds (VOC), nitrogen oxides, sulfur oxide, titanium oxide, lead and its compounds, soot, fumes, ash, and heavy organic compounds in the liquid phase and partially modified as PM particulates (TPM)) (Drzyzga 2004, Xing et al. 2016, Jia et al. 2019). The listed toxic compounds are produced from explosions of various explosives and formulations used in ammunition production (ENVIRO Wiki 2021).

Moreover, very recently, Mariussen et al. (2021) showed that gunshot fumes from firing small arms contain a complex mixture of gases, particles, and aerosols that can be harmful to soldiers, especially lung tissue. The adverse, toxic effects of ammunition explosions and released chemical compounds, especially CO, CO_2_, NO_x_, HCN, NH3, PAH, dioxins, metals, PM2.5, PM10, and unburned energetic materials, were shown to affect also the environment with long-term impact upon military life (Petrea et al. 2018). Chemicals mentioned above are the source of long-term exposure described by health and safety inspectors at workplaces of persons testing the shooting capabilities of new ammunition or people examining the physico-chemical properties of ammunition’s compounds (Campbell et al. 2018). It is worth underlining that the exposure to the specific chemicals is not quantitatively monitored for involved individuals during military training. Toxicity benchmarks are available for many of above-mentioned compounds and combustion products; however, few are derived specifically for military personnel. Assessment of ammunition impact upon its testing is difficult regarding the measurement of the burning products and released waste. Furthermore, the categories of substances found in the firing ranges are carried out different types of trainings and testing activities with armament and ammunition from endowment or being in different stages of development (Johnson 2021).

Due to the inhalation nature of chemical exposure, especially to diphenylamine, volatile organic compounds, and hydrocarbons, and its possible influence on the respiratory tract, a panel of genes and their point mutations correlating with lung oncogenesis was tested using liquid biopsy method. Liquid biopsy is a promising approach for noninvasive assessment of cancer gene profiles and, although it is currently solely used as an alternative when tissue is not available, it can be used to identify mutations occurring in lung cancer both at diagnosis and during the course of disease (Lamy et al. 2020). When tumors develop, small amounts of DNA from the affected cells are present in the patient’s blood and can be detected before clinical symptoms are manifested. The genetic material derived from such cells is different from that released into the peripheral blood by normal cells (Bronkhorst et al. 2019). Unlike traditional tumor tissue biopsies, ccfDNA analysis harbors the potential to detect and monitor the consistently evolving mutational profile of early stage and (oligo-) metastasized cancer.

The presence of detected point mutations as a possible response to long-term toxic compounds exposure was compared with corresponding genes sequences obtained from cheek swab representing congenital genotype. Genetic analysis was performed using the highly advanced MALDI TOF MassARRAY Analyzer 4 (Agena Bioscience, USA) spectrometer with dedicated genes panels, which identifies the presence of point mutations with high sensitivity and accuracy.

### MassARRAY Analyzer 4 platform and mass spectroscopy in the analysis of genetic material polymorphisms

The MassARRAY Analyzer 4 system is a high-quality platform for DNA and RNA analysis, allowing for effective and precise measurement of the amount of genetic material and assessing its variability. MassArray 4 delivers specific and sensitive results based on the combination of a mass spectrometer with a solid molecular biology foundation and advanced data analysis software. The MassARRAY Analyzer 4 system is a system that uses high-performance mass spectrometry (MALDI TOF) technology dedicated to nucleic acids (Griffin and Smith 2001). The first step is the laser desorption of the matrix with which the tested DNA is associated (MALDI, Matrix-assisted Laser Desorption/Ionization). Laser ionization takes place with the beam energy selected in such a way as not to fragment the particles but only to knock them out. Then, the time-of-flight analyzer (TOF) works and the particles introduced into the analyzer are accelerated by an electric pulse and begin to drift through the analyzer chamber. In the end, an ion detector measures time from the accelerating pulse to the moment of impact of a specific molecule on the detector. The measurement of the mass–charge ratio is based on the fact that for a pulse of a given intensity, the flight time for ions increases as their molecular weight increases. The strength of the MassARRAY system is keeping pace with developments in genome-wide studies — mainly in SNP genotyping and quantitative methylation analysis. The system allows obtaining reliable data from complex samples of biological material.

Presented studies showed for the first time that modern research techniques could be implicated as a tool for assessing work safety at selected positions of army soldiers. Obtained preliminary results justify, in our opinion, promising perspective of routine diagnostic of elite professional groups using advanced molecular methods.

## Materials and methods

### Experimental group

Experiments were conducted with the permission of the Local Bioethical Committee (no. 06/2017 and 12/2018). In the first stage of the project, a questionnaire survey was conducted on employees of selected units of land forces employed at workplaces predisposing to contact with harmful inhaled substances, who gave their voluntary consent to participate in a genetic test. The questionnaires contained basic information about the examined person (name, surname, age, job position, and length of service). Each person was marked with a code used in the further stages of the analysis. The survey also asked about smoking and the presence of neoplastic respiratory diseases in the immediate family. Individuals were also examined in the context of activities during which exposure to harmful chemical agents exists and the length of the exposure period at a given workplace. Only persons exposed more than 5 years were selected for analysis. Smoking was an additional criterion determining the exclusion of the respondent from the research. On this basis, a group of people whose work exposure to harmful inhalation factors exceeded five years (33 persons) was selected.

Buccal swabs and a blood sample were obtained from each volunteer. The genetic material obtained from swabs provided information about the innate genetic profile of the volunteers studied. From blood samples, the so-called circulating DNA was isolated. In both samples, point mutation profiles in the selected genes corresponding with lung oncogenesis were analyzed. Obtained profiles were compared with each other, indicating novel, and somatic mutations are absent in the innate genetic profile.

### DNA isolation

Tested subjects did not eat, drink, smoke, or brush their teeth at least one h before sample collecting. To collect a buccal sample, the swab was scraped or brushed against the inside of each cheek for one min. DNA was further isolated using ExtractMe DNA Swab and Semen Kit (Blirt S.A., Gdańsk, Poland). Swab samples were stored at − 20 °C. To isolate DNA, swab holding tissue cells was placed in 1.5-mL Eppendorf tubes, and SSL buffer along with proteinase K were added and vortexed shortly. Swabs were incubated at 56 °C for 40 min and periodically mixed. Next, SSB buffer was added, and swabs were further incubated additional 6 min at 70 °C. Swabs were firmly pressed against one side of the tube in order to retrieve maximum possible volume of lysate and further discard. Next, 99.6% molecular grade ethanol (EtOH) was added, and lysates were transferred onto a purification mini-column and centrifuged for 60 s, 11000* g*. Filtrate was discarded and all remaining lysate was transferred onto purification column. After centrifugation (60 s, 11000* g*), mini-columns have been put into a new collection tubes and washed twice with SSW1 buffer (30 s, 11000* g*). After filtrate discarding, mini-columns were centrifuged for 60 s at 15000* g* and transferred onto a sterile 1.5-mL Eppendorf tube. After addition of elution buffer (previously incubated in 80 °C), mini-columns were incubated for 120 s in RT and centrifuged twice for 60 s at 11000* g*. Isolated DNA was stored at − 20 °C.

For liquid biopsy analysis, blood samples were secured using specialized blood STASIS ™ 21-ccfDNA tubes (MAGBIO Genomics, USA) recommended to analyze circulating nucleic acids and subjected to the isolation procedure using the cfKapture 21 Kit (MAGBIO Genomics, USA) according to the producer’s protocol. Briefly, blood samples were centrifuged (2000* g*, 10 min) and plasma samples were transferred to a canonical tube and centrifuged once again (16000* g*, 10 min) to remove cell debris. Next, to stabilized plasma proteinase K, solution was added and samples were incubated for 10 min at 60 °C. Next, samples were incubated for following 5 min with CFL buffer. After incubation, 100% EtOH, MAG-CFB Particles, and DF solution were added, vortexed, and incubated on a tube rotator for 20 min at RT. Next tubes were placed on a magnetic separation device to magnetize the MAG-CFB Particles for 20 min. After supernatant removing, magnetic particles were resuspended in CWF1 buffer, vortexed 10 times, and transferred to a new 1.5-mL centrifuge tube. The tubes were placed once again in magnetic separation device to magnetize MAG-CFB Particles at RT for 5 min. After incubation, supernatants were removed and the last step was repeated twice using CWF2 buffer. Next, MAG-CFB Particles were resuspended in elution buffer, vortexed, incubated in RT for 10 min, and placed in magnetic separation device for 5 min. Clear supernatants containing the ccfDNA were transferred to a new 1.5-mL tubes and stored at − 20 °C.

Quantification and DNA purity were determined spectrophotometrically using Nabi nano-spectrophotometer (MicroDigital Co., Ltd, Korea) or Qubit™ 4 Fluorometer with dedicated reagents Qubit™ 1X dsDNA HS Assay Kit (Thermo Fisher Scientific, USA).

### Single-nucleotide polymorphisms (SNPs) genotyping

To examine the innate presence of mutations correlated with lung carcinogenesis, DNA isolated from swabs was analyzed with LungCarta or HS Lung Panels (Agena Bioscience, Germany). ccfDNA samples were analyzed with UltraSeek Lung Panel (Agena Bioscience, Germany).

LungCarta panel provides a highly sensitive panel of assays for the evaluation of more than 250 somatic mutations in 26 oncogenes and tumor suppressors for targeted confirmation of genetic events in non-small cell lung tumors. The LungCarta Panel is a compilation of key mutations identified via sequencing discovery studies that affect key pathways in lung adenocarcinoma tumors (Ding and Getz Wilson 2008). The detailed list of point mutations analyzed with LungCarta assay is presented in the Supplementary materials — Table S1. LungCarta assay detects and quantify mutations with frequencies as low as 5%. The HS Lung Panel detects 70 clinically relevant variants across *BRAF*, *EGFR*, *ERBB2*, *KRAS,* and *PIK3CA* genes at as low as 1% variant allele frequency. The detailed list of point mutations analyzed with HS Lung assay is presented in the Supplementary materials — Table S2.

To assess the presence of somatic mutation, ccfDNA was analyzed using UltraSeek Lung Panel (Agena Bioscience, Germany) that detects 70 clinically relevant variants across *BRAF*, *EGFR*, *ERBB2*, *KRAS,* and *PIK3CA* genes at the lowest 0.1% variant allele frequency and is dedicated for liquid biopsy analysis. The detailed list of point mutations analyzed with UltraSeek Lung Panel is presented in the Supplementary materials — Table S3.

The general protocol of DNA samples with presented panels is similar. Each sample is subjected to PCR amplification with dedicated reagents. PCR cocktails contained standard reagents (water, 10xPCR Buffer, MgCl_2_, dUTP/dNTP Mix, PCR Enzyme, PCR Primers. and DNA sample).

Samples were amplified using the following conditions:

**Table:** 

1.95 °C	2 min	1 cycle	
2.95 °C	30 s		
3.56 °C	30 s		
4. 2 °C	1 min		(2–4) 45 cycles
5.72 °C	5 min		1 cycle
6.4 °C	hold		

After amplification, any remaining free deoxynucleotides in the amplification reaction mixtures were dephosphorylated to prevent interference with the further reaction. Shrimp alkaline phosphatase (SAP) dephosphorylates unincorporated dNTPs and converts them to dNDPs, making them unavailable for the subsequent extension reactions. The SAP was then heat inactivated by incubation at 85 °C for 5 min.

Next, the second extension reactions, with dedicated for each panel reagents, were performed under the following conditions:

**Table:** 

1. 95 °C	30 s	1 cycle
2. 95 °C	5 s	
3. 52 °C	5 s	
4. 80 °C	5 s	(3–4) 5 cycles
5. 72 °C	3 min	(2–4) 40 cycles
6. 72 °C	3 min	1 cycle
7. 4 °C	hold	

The primer extension products with the addition of clean resin in the reaction mixture are dispensed onto a SpectroCHIP Array using MassARRAY Nanodispenser and detected via MassARRAY MALDI-TOF mass spectrometry. After the sample run, an automated software report provides the calls and mutations frequency for each sample as well as a confidence score.

## Results

SNPs genotyping revealed the presence of innate mutations (LungCarta and HS Lung Panel), as well as new somatic mutations (UltraSeek Lung Panel) in analyzed genes that appeared de novo during a lifetime. The obtained variants list is summarized below in Table 1.

**Table 1:** The list of detected mutations with LungCarta, HS Lung and UltraSeek Lung Panels, and MassARRAY MALDI-TOF mass spectrometry (Sample ID – letters A, B, C, and D code various military units)

Sample ID	Gene	Detected mutations		
		LungCarta Panel (innate mutations)	HS Lung Panel (innate mutations)	UltraSeek Lung Panel (de novo mutations)
3_1A A8	*BRAF*	G469E	-	-
	*EGFR*	G719R, P753Q	-	-
	*STK11*	V197fs*69	-	-
	*TP53*	R273C	-	-
6_1A A20	*EGFR*	E709K/H	-	-
	*STK11*	Q137*	-	-
7_1A A22	*BRAF*	-	G1406C/T	-
	*EGFR*	E709K/H	-	A2309G, T2573G, L747_A750 > P
	*STK11*	A43_L50del	-	-
	*TP53*	R175H	-	-
10_1A	*EGFR*	-	A2126G	A2309G
11_1A A4	*BRAF*	D594V, D594A, L597V, V600K/M	-	-
	*EGFR*	D770_N77insAPW, G598V, L747_A750del, S752F, E746_S752 > V, R108K	-	-
	*EPHA3*	A435S, N379K	-	-
	*KRAS*	Q61N	-	-
	*MAP2K1*	D67N	-	-
	*MET*	N375S	-	-
	*NRAS*	Q61K	-	-
	*PTPN11*	E76V	-	-
	*PTPRD*	D154Y	-	-
	*STK11*	A43_L50del	-	-
	*TP53*	R282W	-	-
12_1A A23	*BRAF*	L597V, V600K, V600M	-	-
	*EGFR*	N771T, S752F, E746 > S752 > I, E746_S752 > V	2247AtoCr del 19, 2300CtoAr ins20	A2309G
	*MAP2K1*	D67N	-	-
	*NRAS*	Q61K	-	-
	*PTPN11*	E76V	-	-
	*PTPRD*	D164Y	-	-
	*STK11*	Y272Y	-	-
	*TP53*	R273P, R282W	-	-
13_1A	*EGFR*	-	-	A2309G
14_1A A25	*BRAF*	-	-	G1406C/T
	*EGFR*	-	-	L747_A750 >), A2126G, 770_771insSVD
	*KRAS*	-	-	A183C/T
	*TP53*	G245D, R273C	-	-
15_1A A5	*EGFR*	A289V, G719S	-	A2309G
	*NRAS*	Q61K	-	-
18_1A A17	*BRAF*	G469A	-	770_771insG
	*DDR2*	T765P	-	-
	*EGFR*	L858R, E746_T751 > V	-	-
	*KRAS*	Q61H	-	-
	*PTPN11*	E76V	-	-
19_1A	*EGFR*	-	-	A2309G
21_1A	*EGFR*	-	-	A2309G
27_1A	*EGFR*	-	G2390C	L747_A750 > P, T2573G, G2155T/A, L745_E749del
30_1A	*EGFR*	-	T2573G	T2573G, L747_A750 > P, G2155T/A
4_B1	*ALK*	L1196M	-	-
	*DDR2*	G774V	-	-
	*EGFR*	P753S	-	-
	*STK11*	Y272Y	-	-
	*TP53*	R249S	-	-
5_B2	*ALK*	L199M	-	-
	*BRAF*	G469R, V600M	-	-
	*EGFR*	V774L	-	G2155T/A, L747_A750 > P
	*STK11*	Q137*, V197fs*69, V236fs*30	-	-
1_1B2	*EGFR*	P753Q	-	-
	*KRAS*	G12S	-	-
	*MET*	982_1028del47	-	-
	*NOTCH1*	R2328W	-	-
4_1B2	*EGFR*	T790M	-	-
	*EPHA3*	M269I	-	-
	*TP53*	R273C	-	-
3_1C C9	*ALK*	L1196M	-	-
	*BRAF*	V600M	-	-
	*DDR2*	I638F	-	-
	*EGFR*	H773N, H773_V774insNPH, P753S, A289V, D761N	-	T2573G, L747_A750 > P, G2155T
	*KRAS*	G12A	-	-
	*NRAS*	Q61K	-	-
	*STK11*	H174R, V236fs*30	-	-
	*TP53*	R175H, R158C	-	-
5_1C C1	*EPHA3*	M269I	-	-
	*NOTCH1*	R2328W	-	-
	*NTRK3*	V307L	-	-
	*PTEN*	R223*	-	-
	*EGFR*	D770_N771insG, V769_D770insAGGT, D770_D771insAPW	-	A2309G
	*EPHA5*	S556Y	-	-
	*MAP2K1*	Q58P	-	-
	*NTRK2*	G261R	-	-
6_1C C17	*DDR2*	I638F	-	-
	*EGFR*	P772_H773insV	-	T2573G
	*RBB2*	-	-	2324_2325ins(12)ATAC-GTGATGGC
	*KRAS*	G12S/T	-	-
	*STK11*	V236fs*30	-	-
8_1C C15	*ALK*	L1196M	-	-
	*EGFR*	P753S, T751I, R108K	-	A2309G, G2155T, T2573G
	*EPHA3*	W250R	-	-
	*NRF2*	D29H	-	-
12_1C C5	*BRAF*	V600M	-	G1406C/T
	*EGFR*	G719S, T790M	-	A2309G, G2390C
	*KRAS*	-	-	A183C/T
	*ERBB2*	-	2324_2325ins(12)ATACGTGATGGC	-
13_1C C13	*BRAF*	D594V	-	-
	*EGFR*	G719C	-	T2573G, A2126G, L740_750 > P, G2155T
	*KRAS*	G12A	A183C/T	-
	*ERBB2*	-	2324_2325ins(12)ATACGTGATGGC	-
	*PTCH1*	S1326fs*46	-	-
	*NTRK2*	L138F, Q666R	-	-
D6	*EGFR*	-	-	A2309G
D7	*EGFR*	-	-	A2309G, L740_750 > P
	*KRAS*	-	-	A183C/T
	*MET*	982_1028del47	-	-
D8	*MAP2K1*	K57N	-	-
D11	*MET*	N375S	-	-
D12	*DDR2*	C580Y, T765P	-	-
	*EGFR*	-	-	A2309G, L740_750 > P, G2155T/A
	*KRAS*	G12R	-	-
	*STK11*	V23fs*30	-	-
	*NOTCH1*	T1997M	-	-
	*TP53*	R175H, R248G	-	-

fs*—mutation type: insertion-frameshift; *—mutation type: nonsense mutation; the protein coding sequence ends at a translation termination codon (*stop codon*); del—mutation type: deletion; ins—mutation type: insertion

LungCarta and HS Lung Panel revealed the presence of innate point mutations. It has to be explained that LungCarta Panel allows for simultaneous detections of 250 somatic mutations; whereas, HS Lung Panel is dedicated to 70 SNP analyses. Thus, not all mutations detected with the LungCarta Panel were further confirmed with the HS Lung Panel. On the other hand, the sensitivity of the HS Lung Panel is higher and allows detection of genetic variants with frequencies as low as 1%, in comparison to LungCarta (5%). Thus, the HS LungCarta Panel revealed the presence of SNP missed by the LungCarta Panel (sample 12_1A A23).

Most importantly, in 23 out of 29 samples, UltraSeek Panel analysis of ccfDNA revealed the presence of SNPs, in *KRAS*, *EGFR*, *BRAF,* and *ERBB2* genes that were not detected in swab DNA (LungCarta Panel). It indicates that detected mutations appeared de novo. It should be noted also that the UltraSEEK Lung Panel does not include all genes and polymorphisms that can be identified with the LungCarta panel. However, it includes the most important mutations that correlate with the process of lung oncogenesis described in the literature. It should also be noted that the adequate quantities of circulating in the blood DNA is highly lower than the amount of material isolated from swabs. Thus, the threshold of possible detection is critical. The research panels used and the MassArray4 Platform indicate that in several subjects, it is possible to identify circulating DNA containing mutations shown in the panel as factors correlating with the occurrence of neoplastic changes. Bearing in mind the long-term exposure to toxic chemical compound in ammunition, SNPs presence could be the results of their harmful action.

## Discussion

The presence of toxic and VOC constituents from various ammunitions is a known hazardous problem (NPR website 2021, Mariussen et al. 2021). Its long-term exposure could exert various harmful effects on humans, including DNA-damage, oxidative stress, and even neoplastic processes (Mariussen et al. 2021, Mariussen et al. 2021). In our studies, we analyzed selected group of professional soldiers of polish army, as well as other workers, who are engaged in routine ammunition analysis and its explosive properties testing. Because of that, they are exposed to various toxic compounds, such as diphenylamine, carbon monoxide, volatile organic compounds, nitrogen oxides, sulfur oxide, titanium oxide, lead, soot, fumes, ash, and PM particulates (Petrea et al. 2018). During a gunshot, the high amount of above chemicals is released and can be easily inhaled affecting the respiratory tract and lungs. Its negative impact on soldiers has been previously reported by Voie et al. (2014). The authors found that after sessions at shooting ranges with HK416 small arm (Heckler & Koch), soldiers got flu-like symptoms. Further investigations have revealed that exposure to gunshot fumes may induce health effects such as fever, coughing, inflammations reactions, and reduced lung capacity (Voie et al. 2014, Borander et al. 2017). Moreover, the high levels of carbon monoxide in the fume lead to increased levels of carboxyhemoglobin in the blood. On the other hand, CO-poisoning may lead to headache. The authors raised a concern whether a gunshot fumes may induce more long-lasting effects, such as prolonged reduced lung capacity and even cancer. To verify the cancerogenic potential of gushot fumes, Mariussen et al. (2021) performed in vitro studies on human epithelial A549 cell line and examined the DNA damage and cell viability after HK416 gunshot fumes exposure. Obtained results proved that prolonged exposure of A549 cells to gunshot fumes (> 6 shots) induced significant DNA-damage and concomitant reduction of cell viability. The authors concluded as highly probable that repeated exposure to high concentrations of gunshot fumes may induce long-lasting effects, emphasizing the importance of extensive ventilation at training areas and, more importantly, regularly lung function testing of exposed individuals (Mariussen et al. 2021).

Based on presented results, we decided to verify whether prolonged exposure to ammunition-related chemicals could correlate with the appearance of SNPs, known to be correlated with lung carcinogenesis. The conducted research does not have a diagnostic value. However, it constitutes a scientific study that shows that modern research techniques can identify people exposed to harmful inhalation factors at a higher risk of developing their harmful effects. The obtained results showed that most of analyzed “liquid biopsy” (LB) samples revealed the presence of new somatic mutations that were not present in swabs. This information may indicate extended preventive examinations of a particular person or decision about changing the job to one that does not pose an additional health risk. Although the surveyed group of soldiers was not very large, the obtained preliminary results are a good signal that the proposed solution has development potential. We are aware that LB has its methodological and biological limitations that still hamper the implementation of LB into the clinical practice. For example, cell-free DNA could potentially be released from modified cell clones, not having optimal conditions to grow (Heidrich et al. 2021). Thus, in the case of more detailed cancer-related studies, LB should be further verified.

The research carried out under the National Health Program is pioneering. However, it may contribute to developing a protocol for early detection and monitoring of the health of military personnel at workplaces related to the risk of exposure to toxic substances. It may also be extended to other professional groups. MassARRAY offers the possibility of studying various gene panels, not only correlating with lung cancer. Considering the occurring exposures and their possible harmful effects, the undertaken direction of research is promising.

Currently, we are going to extend our research group and follow up on the already analyzed group in the context of their respiratory tract health.

## Supplementary Information

Below is the link to the electronic supplementary material.

Supplementary file1 (DOCX 19 KB)
