# Supplementary material for: Ultrasensitive analysis of genetic instability related to chemical exposure
Source: J Appl Genet. 2021 Dec 30;63(2):305–13. doi: 10.1007/s13353-021-00677-6 (PMC8979864; doi:10.1007/s13353-021-00677-6)
Supplement: Supplementary file 1 — Supplementary file1 (DOCX 19 KB) [file 13353_2021_677_MOESM1_ESM.docx]

**Journal of Applied Genetics**

**Ultrasensitive analysis of genetic instability related to chemical exposure**

Tomasz Domoradzki ^1^, Piotr Grochowski ^2^, Anna Jaśkiewicz ^1^ and Beata Pająk ^1,^*

^1^ Kaczkowski Military Institute of Hygiene and Epidemiology, Kozielska 4, 01-089 Warsaw, Poland; T.D. [domoradzki.tomasz@gmail.com](mailto:domoradzki.tomasz@gmail.com) (ORCID 000-0003-0537-2973)l A.J. [ancpatrin@gmail.com](mailto:ancpatrin@gmail.com) (ORCID 0000-0003-1454-0852)

^2^ Kawaska Sp. z o. o., Zaczarowanej Róży 1, 05-540 Zalesie Górne; Poland [piotr.grochowski@kawaska.pl](mailto:piotr.grochowski@kawaska.pl)

* Correspondence: B.P. [bepaj@wp.pl](mailto:bepaj@wp.pl) (ORCID 0000-0002-3565-8860)

**Table S1.** LungCarta Panel genes and mutations

| **Gene** | **Mutation** |
| --- | --- |
| *AKT1* | E17K |
| *ALK* | C1156Y, L1196M |
| *BRAF* | G469S/E/A/V, D594G/V, L597Q/V, V600E/K/M |
| *DDR2* | L63V, I120M, D125Y, L239R, G253C, G505S, C580Y, I638F, T765P, G774E/V |
| *EGFR* | R108K, T263P, A289V, G598V, E709A/G/V, E709K/H, G719S/C/A/D, E746_T751>A, E746_T751>S, E746_T751>V, E746_S752>A, E746_S752>D, E746_S752>I, E746_S752>V, L747S, L747_E749del, L747_A750del, L747_A750>P, L747_T751>P, L747_S752del, L747_P753>Q, L747_P753>S, A750P, T751A, T751I, T751P, S752F, S752_I759del, P753Q, P753S, D761Y/N, M766_A767insAI, S768I, V769_D770insASV, V769_D770insCV, D770fs*61, D770_N771insAPW, D770_N771insG, D770_N771insGL, D770_N771>AGG/N771>GF, N771T, N771_P772>SVDNR, P772_H773insV, H773N, H773_V774insNPH/PH/H, V774L, V774_C775insHV, R776C/H, T790M, T854A, L858R/M, L861Q/L861R |
| *EPHA3* | T37K, N85S, T166N, G187R, S229Y, W250R, M269I, N379K, T393K, A435S, D446Y, S449F, G518L, K761N, G766E, D806N |
| *EPHA5* | D493Y, S566Y, G582E, S810I, T856I, R1007Q, N1032S, M1034I |
| *ERBB2* | M774_A775insAYVM, A775_G776insAYVM |
| *FGFR4* | H192fs*19, P672T |
| *JAK2* | P503L, L609S, Y931C, R1122P |
| *KRAS* | G12S/V/F/R/A/C/D, G13C/S/A/V/D, Q61L/R/P/H/E/K |
| *MAP2K1* | Q56P, K57N, D67N |
| *MET* | N375S, 982_1028del47 |
| *NOTCH1* | D1643H, V1672I, T1997M, H2276fs*79, R2328W, V2444fs*35 |
| *NRAS* | Q61E/K/H/L/R/P |
| *NRF2* | D29H, G31A, R34Q, D77N/A, E79Q/K/G, G81D |
| *NTRK1* | Q80*, R119H, S326R |
| *NTRK2* | C45F, L138F, G261R, Q666R, L670M, L755L |
| *NTRK3* | L152I, S184C, L248M, L270M, T283K, V307L, L336Q, R721G, I769N |
| *PIK3CA* | E542Q/K, E545Q/K, H1047Y/R/L |
| *PTCH1* | R682L, R1308G, S1326fs*46 |
| *PTEN* | R233* |
| *PTPN11* | E76V |
| *PTPRD* | T337A, V483E, S1703R |
| *STK11* | I26fs*25, Q37L, A43_L50del6, L50_D53del4, M51fs*14, G56fs*4, G56W, E70*, E70fs*26, K78E, R86G, G91L, E120*, Q123R, Q137*, Q159*, G163C, E165*, Q170*, H174R, P179L, G188fs*99, K191*, G196V, V197fs*69, Q220*, E223*, V236fs*30, Y272Y, L285Q, D327fs*10, A347fs*13, F354L, R426W |
| *TP53* | V157F, R158C/G/L/P, Y163C, R175L/H, Y220C, G245C/S, G245D/V, R248G/L/Q/W, R249S/W/M, R273C/H/L/P, R282G/W |

**Table S2.** HS Lung Panel genes and mutations

| **Gene** | **Mutation** |
| --- | --- |
| *BRAF* | c.1406G>T, c.1406>C, c.1781A>G, c.1799T>A |
| *EGFR* | c.2240_2251del(12)TAAGAGAAGCAA, c.2582T>A c.2239_2247del(9)TTAAGAGAA c.2238_2255del(18)ATTAAGAGAAGCAACATC, c.2235_2249del(15)GGAATTAAGAGAAGC c.2573T>G, c.2236_2250del(15)GAATTAAGAGAAGCA c.2156G>C, c.2369C>T, c.2303G>T, c.2155G>A, c.2155G>T, c.2239_2256del(18)TTAAGAGAAGCAACATCT, c.2237_2254del(18)AATTAAGAGAAGCAACAT, c.2240_2254del(15)TAAGAGAAGCAACAT, c.2240_2257del(18)TAAGAGAAGCAACATCTC, c.2126A>T, c.2582T>G, c.2307_2308ins(9)GCCAGCGTG, c.2319_2320InsCAC, c.2310_2311insGGT, c.2319_2320ins(9)AACCCCCAC, c.2239_2248TTAAGAGAAG>C, c.2239_2251TTAAGAGAAGCAA>C, c.2237_2255>T, c.2237_2252>T, c.2239_2258>CA, c.2239_2256>CAA, c.2237_2253>TTGCT, c.2238_2248>GC, c.2308_2309ins(9)CCAGCGTGG, c.2237_2251del(15)AATTAAGAGAAGCAA, c.2236_2253del(18)GAATTAAGAGAAGCAACA, c.2125G>A, c.2126A>G, c.2126A>C, c.2311_2312ins(9)GCGTGGACA, c.2235_2248>AATTC, c.2235_2251>AATTC, c.2309_2310AC>CCAGCGTGGAT, c.2237_2257>TCT, c.2311_2312ins(3)CAC, c.2235_2246del(12)GGAATTAAGAGA, c.2237_2253>TTCCT, c.2390G>C, c.2389T>A |
| *ERBB2* | c.2326_2327ins(3)TTT, c.2326_2327ins(3)TGT, c.2325_2326ins(12)TACGTGATGGCT, c.2324_2325ins(12)ATACGTGATGGC |
| *KRAS* | c.34G>T, c.34G>A, c.34G>C, c.35G>T, c.35G>A, c.35G>C, c.34_35GG>TA, c.37G>T, c.38G>A, c.181C>A, c.181C>G, c.182A>C, c.182A>G, c.182A>T, c.183A>C, c.183A>T |
| *PIK3CA* | c.1624G>A, c.1633G>A, c.3140A>G, c.3140A>T |

**Table S3.** UltraSeek Lung Panel genes and mutations

| **Gene** | **Mutation** |
| --- | --- |
| *BRAF* | c.1406G>T, c.1406>C, c.1781A>G, c.1799T>A |
| *EGFR* | c.2240_2251del(12)TAAGAGAAGCAA, c.2582T>A c.2239_2247del(9)TTAAGAGAA c.2238_2255del(18)ATTAAGAGAAGCAACATC, c.2235_2249del(15)GGAATTAAGAGAAGC c.2573T>G, c.2236_2250del(15)GAATTAAGAGAAGCA c.2156G>C, c.2369C>T, c.2303G>T, c.2155G>A, c.2155G>T, c.2239_2256del(18)TTAAGAGAAGCAACATCT, c.2237_2254del(18)AATTAAGAGAAGCAACAT, c.2240_2254del(15)TAAGAGAAGCAACAT, c.2240_2257del(18)TAAGAGAAGCAACATCTC, c.2126A>T, c.2582T>G, c.2307_2308ins(9)GCCAGCGTG, c.2319_2320InsCAC, c.2310_2311insGGT, c.2319_2320ins(9)AACCCCCAC, c.2239_2248TTAAGAGAAG>C, c.2239_2251TTAAGAGAAGCAA>C, c.2237_2255>T, c.2237_2252>T, c.2239_2258>CA, c.2239_2256>CAA, c.2237_2253>TTGCT, c.2238_2248>GC, c.2308_2309ins(9)CCAGCGTGG, c.2237_2251del(15)AATTAAGAGAAGCAA, c.2236_2253del(18)GAATTAAGAGAAGCAACA, c.2125G>A, c.2126A>G, c.2126A>C, c.2311_2312ins(9)GCGTGGACA, c.2235_2248>AATTC, c.2235_2251>AATTC, c.2309_2310AC>CCAGCGTGGAT, c.2237_2257>TCT, c.2311_2312ins(3)CAC, c.2235_2246del(12)GGAATTAAGAGA, c.2237_2253>TTCCT, c.2390G>C, c.2389T>A |
| *ERBB2* | c.2326_2327ins(3)TTT, c.2326_2327ins(3)TGT, c.2325_2326ins(12)TACGTGATGGCT, c.2324_2325ins(12)ATACGTGATGGC |
| *KRAS* | c.34G>T, c.34G>A, c.34G>C, c.35G>T, c.35G>A, c.35G>C, c.34_35GG>TA, c.37G>T, c.38G>A, c.181C>A, c.181C>G, c.182A>C, c.182A>G, c.182A>T, c.183A>C, c.183A>T |
| *PIK3CA* | c.1624G>A, c.1633G>A, c.3140A>G, c.3140A>T |
